# Treatment options for infected bone defects in the lower extremities: free vascularized fibular graft or Ilizarov bone transport?

**DOI:** 10.1186/s13018-020-01907-z

**Published:** 2020-09-24

**Authors:** Gao-hong Ren, Runguang Li, Yanjun Hu, Yirong Chen, Chaojie Chen, Bin Yu

**Affiliations:** 1 Division of Orthopaedics and Traumatology, Department of Orthopaedics, Nanfang Hospital, Southern Medical University, Guangzhou, China; 2 Key Laboratory of Bone and Cartilage Regenerative Medicine, Nanfang Hospital, Southern Medical University, Guangzhou, China; 3 Department of Orthopedics, Third Affiliated Hospital of Southern Medical University, Guangzhou, China; 4 Orthopaedic Hospital of Guangdong Province, Guangzhou, China; 5 Academy of Orthopaedics, Guangdong Province, Guangzhou, China; 6 Department of Orthopedics, Linzhi people’s hospital, Linzhi, China; 7 Department of Orthopedics, Panyu Hospital of Chinese Medicine, Guangzhou, China

**Keywords:** Bone defect, Bone infection, Ilizarov bone transport, Free vascularized fibular graft

## Abstract

**Abstract:**

**Objective:**

The objective was to explore the relative indications of free vascularized fibular graft (FVFG) and Ilizarov bone transport (IBT) in the treatment of infected bone defects of lower extremities via comparative analysis on the clinical characteristics and efficacies.

**Methods:**

The clinical data of 66 cases with post-traumatic infected bone defects of the lower extremities who underwent FVFG (*n* = 23) or IBT (*n* = 43) from July 2014 to June 2018 were retrieved and retrospectively analyzed. Clinical characteristics, operation time, and intraoperative blood loss were statistically compared between two groups. Specifically, the clinical efficacies of two methods were statistically evaluated according to the external fixation time/index, recurrence rate of deep infection, incidence of complications, the times of reoperation, and final functional score of the affected extremities.

**Results:**

Gender, age, cause of injury, Gustilo grade of initial injury, proportion of complicated injuries in other parts of the affected extremities, and numbers of femoral/tibial defect cases did not differ significantly between treatment groups, while infection site distribution after debridement (shaft/metaphysis) differed moderately, with metaphysis infection little more frequent in the FVFG group (*P* = 0.068). Femoral/tibial defect length was longer in the FVFG group (9.96 ± 2.27 vs. 8.74 ± 2.52 cm, *P* = 0.014). More patients in the FVFG group presented with moderate or complex wounds with soft-tissue defects. FVFG treatment required a longer surgical time (6.60 ± 1.34 vs. 3.12 ± 0.99 h) and resulted in greater intraoperative blood loss (873.91 ± 183.94 vs. 386.08 ± 131.98 ml; both *P* < 0.05) than the IBT group, while average follow-up time, recurrence rate of postoperative osteomyelitis, degree of bony union, and final functional scores did not differ between treatment groups. However, FVFG required a shorter external fixation time (7.04 ± 1.72 vs. 13.16 ± 2.92 months), yielded a lower external fixation index (0.73 ± 0.28 vs. 1.55 ± 0.28), and resulted in a lower incidence of postoperative complications (0.87 ± 0.76 vs. 2.21±1.78, times/case, *P* < 0.05). The times of reoperation in the two groups did not differ (0.78 ± 0.60 vs. 0.98 ± 0.99 times/case, *P* = 0.615).

**Conclusion:**

Both FVFG and IBT are effective methods for repairing and reconstructing infected bone defects of the lower extremities, with unique advantages and limitations. Generally, FVFG is recommended for patients with soft tissue defects, bone defects adjacent to joints, large bone defects (particularly monocortical defects), and those who can tolerate microsurgery.

Traumatic open bone injury is often accompanied by infection, which dramatically complicates correction of bone defects and restoration of function [1, 2]. Before the reconstruction of bone defects, more thorough debridement is needed, including the application of various debridement techniques [1]. Free vascularized fibular graft (FVFG) and Ilizarov bone transport (IBT) have been considered as two classic and effective reconstruction techniques for treating bone defects of the extremities. In recent years, substantial advances in Ilizarov technique and related devices have been introduced, such as bone shortening–lengthening [3], double-level bone transport [4], L-shaped corticotomy with bone flap sliding [5], internal fixation-assisted bone transport [6], replacement of internal fixation at the late stage of bone transport [7], inclusion of antibiotic (calcium sulfate) in bone defect and space occupying technique [1], the accordion maneuver [8], and prevention and management of pin track sepsis [9], among others. These techniques can shorten the time required for IBT and external fixation, thereby effectively reducing the incidence of postoperative mechanical line deviation and pin track infection, as well as promoting superior bony union at the docking sites. Consequently, use of IBT has increased for the treatment of bone defects compared with FVFG.

The Masquelet induced membrane technique [10] is another more recent method for the treatment of bone defects. However, this technique requires secondary surgery, making it difficult to treat soft tissue defects. Thus, FVFG and IBT are currently considered appropriate options, but the better choice for lower extremity infected bone defects of various types, locations, ranges, and degrees remains unclear. In addition to subjective factors such as the willingness of patients and surgeons, what clinical characteristics can be considered as the basis for the selection between FVFG and IBT? In fact, there are few direct comparisons of these techniques, likely because they belong to different surgical research fields (microsurgery versus external fixator repair and reconstruction). In 2001, Yokoyama et al. [11] reported no difference in external fixation time, complication rate, hospital costs, union rate, or functional score between FVFG and IBT (*n* = 4 for each group) for the treatment of post-traumatic tibial defects. Alternatively, El-Gammal et al. [12] suggested that the Ilizarov technique yielded greater efficacy for the management of traumatic tibial deficits less than 12 cm in length, whereas FVFG was more efficacious when the tibial deficit was 12 cm or more. To identify the relative indications FVFG and IBT, we retrospectively compared clinical features and outcome metrics (external fixation time, complication rate, union rate, and functional scores) between 66 patients with infected femoral/tibial bone defects treated from July 2014 to June 2018.

## Materials and methods

### Inclusion and exclusion criteria

The study protocol was approved by the ethics committee of Nanfang Hospital (affiliated with Southern Medical University, Guangzhou, China). In this retrospective non-randomized controlled study, patients received the indicated surgery according to baseline clinical condition (systemic and local), personal choice, and/or the surgeon’s judgment. All procedures were performed by surgeons from our operation team. Inclusion criteria were (1) age 16–65 years, (2) femoral/tibial bone defects > 6 cm (including longitudinal defects), (3) infection at the bone defects (as evidenced by topical redness and swelling, hot, pain, sinus formation or pyorrhea, exposure of bone or internal fixator, significantly elevated inflammatory cytokine levels, positive bacteriological test, or pathological diagnosis) [13], and (4) annular bone defects after debridement requiring fixation. Exclusion criteria were (1) lost to follow-up and (2) intolerance to either procedure. Eventually, 66 eligible patients were recruited according to these inclusion and exclusion criteria. Among them, 23 cases received FVFG, and 43 cases received IBT.

### Treatment methods

#### Surgical methods

##### Removal of lower extremity osteomyelitis lesions

The site and extent of the infected lesions were evaluated, and tissue samples were collected for bacteriological culture. As described in our previous methods [14–17], infected and inactivated tissues in the lesions were thoroughly eradicated; the patency of the medullary cavity was restored; dead and slerotic bones were removed until blood oozed from the bone surface (red pepper sign); and the infected periosteum and surrounding inflammatory soft tissues were removed. Depending on the eradication of infection, relatively healthy bone was retained in the FVFG group, while bilateral ends of the bone defects were trimmed in the IBT group.

##### FVFG group

A contralateral fibular flap (an ipsilateral fibula flap could also be used for femoral defect) 2–5-cm longer than the bone defect (8–18 cm, 12.8 cm on average) was designed and harvested according to the methods proposed by Wei and colleagues [18, 19]. For patients with soft tissue defects or poor soft tissue condition, the fibular flap was harvested with a skin flap of appropriate size. Following eradication of the infected lesions, an external fixator was utilized to stabilize the femur or tibia, and the mechanical line and length were adjusted. A bone slot 1.5–2.0-cm wide and 2.0–3.0-cm long was created at one end of the femur or tibia defect site. According to our previously reported methods [16], one end of the repaired fibular flap was inserted into the proximal or distal medullary cavity of the bone defect, while the other end was fixed in the bone slot to bridge the defect. The muscle tissues attached to the fibular flap were then embedded into the infected dead cavity, and one of the fibular ends at the recipient slot site was fixed together with the femur or tibia by hollow screws or Kirschner wires. Finally, the nutrient vessels of the fibular flap on one end of the fibula were anastomosed with healthy vessels in the recipient site, while vascular defects were repaired by vein graft if necessary. The external fixator was then stabilized across the bone defects. Finally, the nutrient vessels of the fibular flap on the one end of the fibula was anastomosed with the healthy vessels in the recipient site; meanwhile, vascular defect needed to be repaired by vein graft [16]. The external fixator was then stabilized across the bone defects.

##### IBT group

Depending on the fracture site and severity of bone defects, a unilateral, circular, or combined unilateral plus circular external fixator (OrthoFix, Italy or Tianjin Xinzhong Medical Devices Co., Ltd.) was selected. During the fixation process, the mechanical line was adjusted to avoid rotation and angulation. The fixation method and needle insertion path were the same as described by Nayagam [20]. Briefly, the two ends of the bone defect were trimmed, and limb shortening was performed as needed (which also reduced tension on sutures at sites of soft tissue damage). Osteotomy was performed at the proximal or distal end of the femur/tibia. Postoperatively, bone sliding was conducted at an appropriate speed. IBT was allowed to proceed toward the two ends, and then limb lengthening was conducted. Wounds were treated by direct suture, local flap transfer, surgery only (open IBT), or early-stage free flap graft.

#### Postoperative treatment

In the FVFG group, patients were carefully monitored according to the institutional management protocol after microsurgery. Patients received anti-infection, anti-spasm, and anti-coagulation therapies as needed and were administered sedative and analgesic agents to relieve vasospasm. Nutritional and supportive treatments were also provided. The blood flow of the skin flap was carefully monitored to insure timely management of vascular crises. Once survival of the skin flap was assured, patients were allowed to perform flexion and extension activities at adjacent joints. Patients were instructed to gradually restore weight-bearing while walking as healing of the bone ends progressed.

In the IBT group, bone transport and sliding were performed according to the classic method proposed by Ilizarov GA [21]. The distraction rate was 0.5–1 mm/day and the distraction frequency was 2–4 times/day. X-ray examination was performed on a regular basis. The bone transport speed was adjusted according to the conditions of the new bony callus. Any deviation of the mechanical line was corrected in a timely manner. After contact of the two fracture ends, healing was carefully monitored. If the contact site was small, bone grafting was immediately conducted, while if the contact site was large, pressure and the Accordion maneuver [8] were applied to promote healing. The external fixator was removed if continuous cortex was revealed on at least three sides of the fracture end by X-ray imaging, bone density had recovered to a normal level, and there was no obvious discomfort during weight-bearing while walking 1 week after the external frame was loosened [22].

### Clinical characteristics analysis and efficacy evaluation

#### Analysis of clinical characteristics

The following clinical characteristics were compared between surgical treatment groups: gender, age, cause of injury, Gustilo grade of initial injury, proportion of complicated injuries in other parts of the affected extremity, bone defect site (shaft/metaphysis) after debridement, bone defect length, and treatment of soft tissue defects.

#### Surgical procedures and efficacy evaluation

The operation time and intraoperative blood loss were statistically compared between two groups. During postoperative follow up, surgical efficacy between two groups was evaluated: (1) incidence or recurrence rate of deep infection, (2) bony union, (3) external fixation time and external fixator index, (4) functional score of the affected extremities (application of the method of Ilizarov, ASAMI [23]), (5) incidence of complications (modified from El-Gammal et al. [12]), and (6) the times of reoperation. Complications in each group were categorized into minor, moderate, and major as illustrated in Table 1.

**Table 1:** Complications are categorized according to the method of El-Gammal et al. with slight modifications

Complications	Free vascularized fibular graft	IIizarov bone transport
Minor	Superficial infection,	Superficial infection,
	Bony malunion,	Bony malunion,
	Grades I and II pin tract reaction,	Grades I and II pin tract reaction,
	Temporary joint stiffness	Temporary joint stiffness,
		Mechanical line deviation during bone
		transport,
		Delayed union of bone contract ends
Moderate	Flap vascular crisis,	Grade III nail tract reaction,
	Grade III nail tract reaction,	Severe mechanical line deviation,
	Severe mechanical line deviation,	Bony nonunion,
	Bony nonunion,	Re-fracture,
	Re-fracture,	Osteomyelitis recurrence,
	Osteomyelitis recurrence	Malreduction at docking site
Major	Severe joint stiffness,	Severe joint stiffness,
	Limb shortening,	Limb shortening,
	Final mechanical line deviation	Final mechanical line deviation

Complications in each group were divided into minor, moderate, and major categories. Minor complications are the complications that require no operative treatment (e.g., pin tract infection). Moderate complications are the complications that require operative treatment (e.g., nonunion). Major complications are the residual complications that could not be corrected (e.g., residual shortening and joint contracture)

### Statistical analysis

All statistical analyses were conducted using IBM SPSS software (Version 20.0., Armonk, NY, IBM Corp.). Continuous variables are expressed as mean ± standard deviation and dichotomous variables as percentage. Group means and proportions were compared by non-parametric test ( Mann–Whitney) and chi-square test, respectively. A *P* < 0.05 (two-tailed) was considered statistically significant for all tests.

## Results

### Distinct clinical characteristics of FVFG and IBT treatment groups

The baseline clinical data of FVFG and IBT groups are summarized in Table 2. There were no group differences in sex ratio (male/female: 16/7 vs. 28/15), mean age (36.13 ± 12.61 vs. 37.35 ± 13.20 years), cause of injury (traffic accident/fall/crush: 14/5/4 vs. 29/9/5), Gustilo grade of the initial injury (closed fracture/Gustilo grade I/Gustilo grade II/Gustilo grade III: 2/3/4/14 vs. 3/4/6/30), proportion of complicated injuries in other parts of the affected extremities (6/23 vs. 11/43), and ratio of femoral to tibial defect cases (5/18 vs. 11/32). Shaft infection was a little more frequent than metaphysis infection in the Ilizarov group (32/11) but not the FVFG group (12/11) (*P* = 0.068), and femoral/tibial defect length was longer in the FVFG group (9.96 ± 2.27 vs. 8.74 ± 2.52 cm, *P* = 0.014). Notably, the treatment choice for soft tissue defects differed significantly between groups (*P* = 0.031). In addition, moderate or complex wounds were more frequent in the FVFG group compared with the IBT group.

**Table 2:** Comparison of clinical data of patients between the free vascularized fibular graft and IIizarov bone transport groups

Variables	Free vascularized fibular graft group	IIizarov bone transport group	*P* value
*n*	23	43	/
Gender			
Male	16	28	0.467
Female	7	15	
Age	36.13 ± 12.61 (years)	37.35 ± 13.20 (years)	0.747
Cause of injury			
Traffic accident injury	14	29	0.791
Falling injury	5	9	
Crush injury	4	5	
Gustilo grade			
Closed fracture	2	3	0.908
Gustilo grade I	3	4	
Gustilo grade II	4	6	
Gustilo grade III	14	30	
Number of complicated injuries in other parts of the affected extremity	6	11	0.964
Number of femoral/tibial defect cases	5/18	11/32	0.729
Femoral/tibial defect and infection site			
Shaft	12	32	0.068
Metaphysis (the distance between lesions and joint surface is ≤ 3 cm)	11	11	
Femoral/tibial defect length after debridement (including longitudinal defects) (cm)	9.96 ± 2.27	8.74 ± 2.52	0.014
Management of different types of soft-tissue defects			
Minor wounds can be repaired by direct suture, skin grafting and local flap transfer.	7	27	0.031
Moderate wounds can be repaired by free vascularized fibular graft with flap or open Ilizarov bone transport.	12	10	
Major wounds require simultaneous or staged free flap graft.	4	6	

### Surgical procedures and efficacy evaluation

As illustrated in Table 3, operation time was significantly longer in the FVFG group than the IBT group (6.60 ± 1.34 vs. 3.12 ± 0.99 h, *P* < 0.05). In addition, intraoperative blood loss was significantly greater in the FVFG group (873.91 ± 183.94 vs. 386.05 ± 131.98 ml, *P* < 0.05). In contrast, average follow-up time did not differ (31.83 ± 7.77 vs. 34.14 ± 7.11 months, *P* = 0.175). Osteomyelitis recurred after operation in two FVFG patients and three IBT patients (8.70% vs. 6.98%, *P* = 1.0), all requiring further debridement and implantation of antibiotic-containing bone meal (calcium sulphate). FVFG required a shorter external fixation time (7.04 ± 1.72 vs. 13.16 ± 2.92 month; *P* < 0.05) and yielded a lower external fixation index (0.73 ± 0.28 vs. 1.55 ± 0.28, *P* < 0.05).

**Table 3:** Surgical procedures and efficacy evaluation between the free vascularized fibular graft and Ilizarov bone transport groups

Variables	Free vascularized fibular graft	Ilizarov bone transport	*P* value
*N*	23	43	/
Operation time (h)	6.60 ± 1.34	3.12 ± 0.99	< 0.001
Intraoperative blood loss (ml)	873.91 ± 183.94	386.05 ± 131.9	< 0.001
Follow-up time (month)	31.83 ± 7.77	34.14 ± 7.11	0.175
Number of cases of deep infection or osteomyelitis recurrence	2	3	1.000
External fixation time (month)	7.04 ± 1.72	13.16 ± 2.92	< 0.001
External fixator index (%)	0.73 ± 0.28	1.55 ± 0.28	< 0.001
Fracture healing evaluation			
Excellent (*n*)	15	25	0.905
Good (*n*)	3	8	
Fair (*n*)	2	3	
Poor (*n*)	3	7	
Excellent and good fracture healing rate	78.26%	76.74%	0.617
Extremity functional evaluation			
Excellent (*n*)	11	21	0.901
Good (*n*)	8	13	
Fair (*n*)	3	8	
Poor (*n*)	1	1	
Excellent and good extremity functional rate (%)	82.61%	79.01%	0.471
Incidence of postoperative complications (time/case)			
Minor	0.22 ± 0.42	1.02 ± 0.99	0.001
Moderate	0.48 ± 0.59	0.88 ± 0.91	0.085
Major	0.17 ± 0.39	0.30 ± 0.46	0.259
Total	0.87 ± 0.76	2.21 ± 1.78	0.001
Reoperation(times/case)	0.78 ± 0.60	0.98 ± 0.99	0.615

Criteria for bone results:

Excellent: union, no infection, deformity < 7°, limb length discrepancy (LLD) < 2.5 cm

Good: union + any two of the following: absence of infection, deformity < 7°, LLD < 2.5 cm

Fair: union + any one of the following: absence of infection, deformity < 7°, LLD < 2.5 cm

Poor: nonunion/refracture/union + infection + deformity > 7°+ LLD > 2.5 cm

Criteria for functional results:

Excellent: active, no limp, minimum stiffness (loss of < 15° knee extension/ < 15° ankle dorsiflexion) no reflex sympathetic dystrophy (RSD), insignificant pain

Good: active, with one or two of the following: limb, stiffness, RSD, significant pain

Fair: active, with three or all of the following: limb, stiffness, RSD, significant pain

Poor: inactive (unemployment or inability to return to daily activities because of injury)

Failure: amputation

We used the average number of reoperations per patient (times/case) as the evaluation index in the two groups. One reoperation may be due to a single complication or multiple complications; on the other hand, some complications may require two or more reoperations

**Fig. 1:**
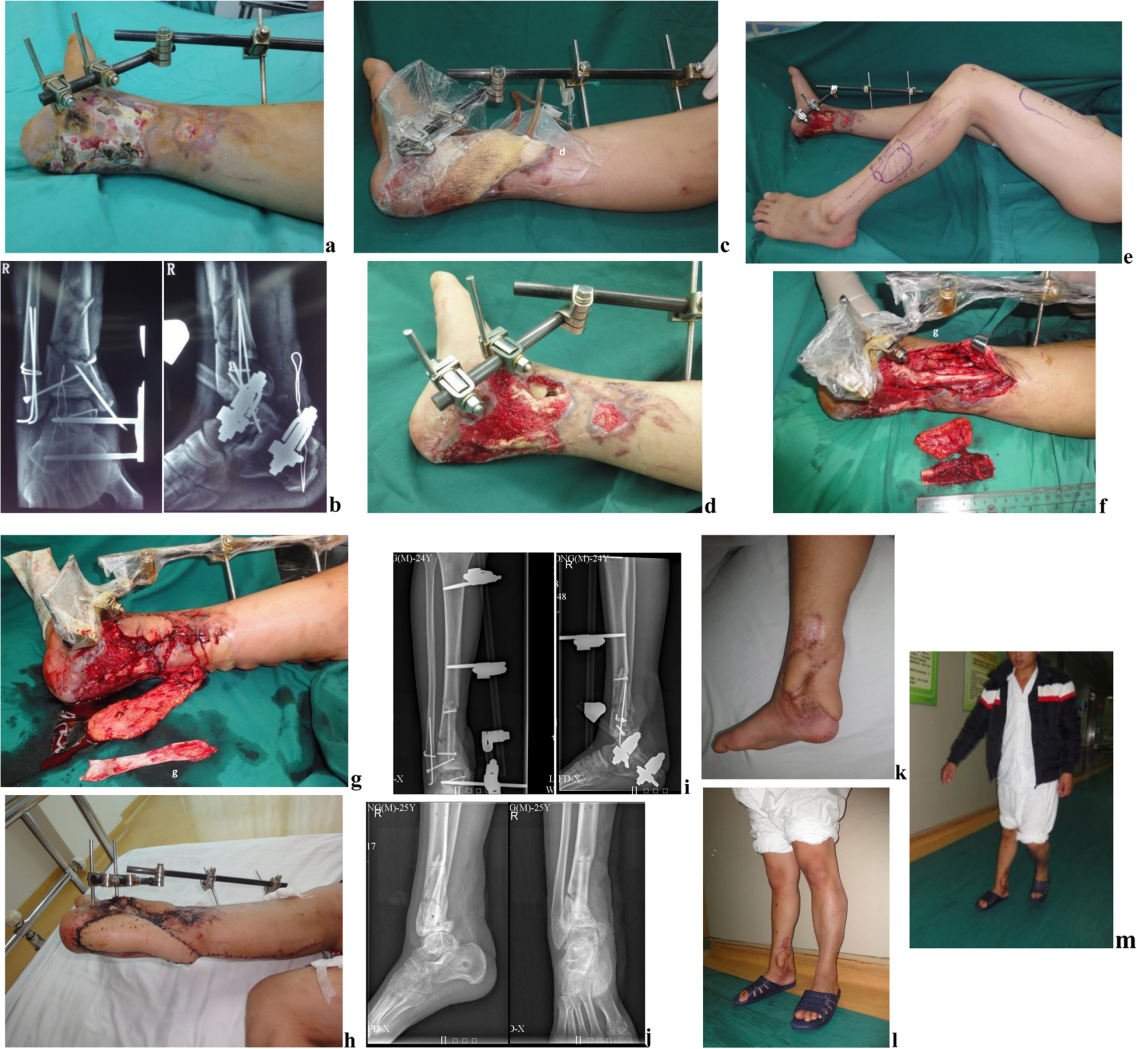
Case 1. A 24-year-old male patient with multiple fractures of the distal tibia and fibula complicated with soft tissue defects and infection for more than 1 month. Injury was caused by a traffic accident. Bone injury appearance upon admission and X-ray of the ankle joint (**a**, **b**). After thorough debridement and vacuum sealing drainage (VSD) of the right ankle, the granulation tissues on the wound surface grew well (**c**, **d**). During the second-stage procedures, FVFG was performed to reconstruct the infected bone defects (approximately 6 cm in size) at the distal tibia. The fascia lata of the same thigh was designed to repair the Achilles tendon defects. Simultaneously, the sural neurovascular flap of the right limb was reversely transferred to repair the Achilles tendon wound. The contralateral fibular bone flap, thigh fascia lata, and the ipsilateral sural neurovascular flap were harvested (**e**–**g**). Both the fibular flap and sural neurocutaneous flap survived well, and the wound was healed without exudation after operation (**h**). Postoperative X-ray showed that the FVFG repaired the distal tibial defects with excellent alignment (**i**). External fixator was removed 6 months postoperative and partial weight-bearing walk under the protection of the brace. At 1 year after operation, the internal fixator was removed, and normal walking function was restored. (**j**–**m**). After 24 months of postoperative time, the ASAMI functional score of the affected extremity was excellent, with an external fixation index 1.0

**Fig. 2:**
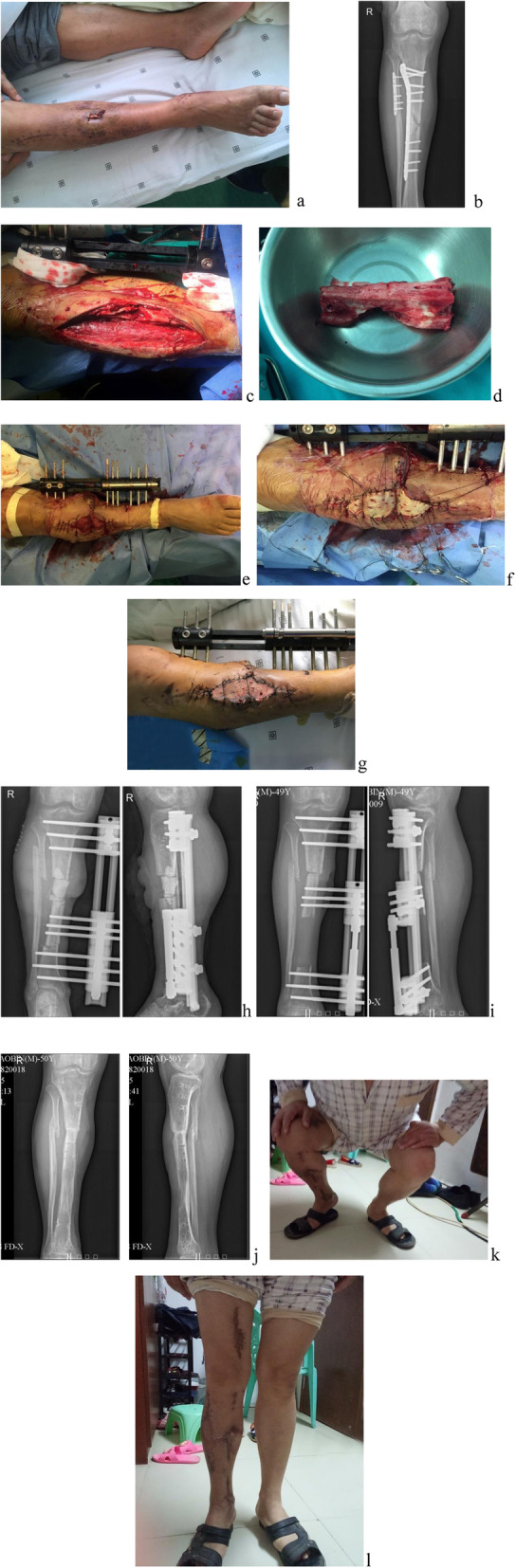
Case 2. A 49-year-old male patient with multiple open fractures of the right tibia and fibula (Gustilo grade II) due to a traffic accident underwent emergency debridement and internal fixation in a local hospital for half a year. Deep infection occurred after the operation, which was still uncontrollable after three times of debridement. Sinus tract was observed on the medial side of the lower extremity with pus (**a**). X-ray showed the tibia and fibula defect and sclerosis in part edge (**b**). Extended debridement, internal fixator removal, infected bone segment excision (approximately 9 cm), external fixation, tibiofibular shortening, distal tibial osteotomy, and full-thickness skin grafting were performed (**c**–**f**). At 1 week after operation, the wounds healed well, and the skin graft survived (**g**). Postoperative X-ray showed the tibial defects with good alignment (**h**). At 4 months after bone transport, the bone ends were contacted and the patient began weight-bearing walk (**i**). At 13 months after the operation, bone fracture healed well, and the external fixator was removed to restore normal walking function. (**j**–**l**). After 24 months of operation, the ASAMI functional score of the affected extremity was excellent, with an external fixation index 1.44

The degree of bony union and functional recovery did not differ between treatments. The proportions of patients with excellent, good, fair, and poor fracture healing were similar (15/3/2/3 vs. 25/8/3/7, respectively, *P* = 0.905; excellent/good: 78.26% vs. 76.74%, *P* = 0.617), as were the proportions showing excellent, good, fair, and poor final functional outcome (11/8/3/1 vs. 21/13/8/1, respectively, *P* = 0.901; excellent/good: 82.61% vs. 79.01%, *P* = 0.471). However, postoperative complications were less frequent in the FVFG group (0.87 ± 0.76 vs. 2.21 ± 1.78, times/case, *P* < 0.05). The times of reoperation in the two groups did not differ (0.78 ± 0.60 vs. 0.98 ± 0.99 times/case, *P* = 0.615). Typical cases are shown in Figs. 1 and 2.

## Discussion

The advantages and disadvantages of FVFG and IBT are well documented [1, 12, 16, 24–26]. In recent years, the IBT technique has gained popularity for the treatment of infected bone defects in the lower extremities compared with FVFG. However, this retrospective study demonstrates that FVFG still play an irreplaceable role for certain infected bone defect cases. There were no significant group differences in gender, age, cause of injury, Gustilo grade of initial injury, frequency of complicated injuries in other parts of the affected extremity, ratio of femoral to tibial defect cases, bone infection site after debridement, while the length of femoral or tibial defects and the number of patients with moderate or complex wounds were significantly higher in the FVFG group. We believe that this difference can be explained by the distinct volume alterations during these two surgeries. FVFG is a volume-increasing operation as it requires better soft tissue coverage to increase the operation rate of the skin flap. In contrast, IBT is a volume-reducing procedure that allows local tissue exposure after bone debridement to decrease the operation rate of local or free flaps. Accordingly, based on this clinical data, we believe that the comparative evaluation of preoperative status, intraoperative status, and clinical efficacy between treatment groups can help in determining the more appropriate treatment. The recurrence rate of osteomyelitis, fracture healing rate, excellent extremity function rate in the advanced stage and the times of reoperation did not differ between groups. Alternatively, external fixation time, external fixator index, and incidence of postoperative complications were lower in the FVFG group, whereas operation time and intraoperative blood loss were higher in this group compared with the IBT group. Thus, while FVFG has certain advantages, it may entail greater surgical risk. The surgical indications should be selected with caution according to the general condition of the patient and technical proficiency of the surgeons in microsurgery.

Given these advantages and disadvantages, it is critical to identify those cases more suitable for FVFG. Although the distribution of bone defect infection sites after debridement (shaft or metaphysis) did not differ significantly between groups (*P* = 0.068), there was a strong trend for more frequent metaphysis infection in the FVFG group. There were significantly longer femoral/tibial defects (*P* = 0.014) after debridement in the FVFG group. Based on previous findings [3, 11, 12, 16, 19, 25, 26] with our experience, we suggest that FVFG is the better option for patients with the following clinical factors. First, patients with infected lesions and defects located at or adjacent to the epiphyseal end and articular surface are good candidates for FVFG. The residual bone volume is relatively small after debridement, making it difficult to install a portable external fixator and prolonging the time required to install a transarticular external fixator. However, the fibular graft and the residual femoral/tibial bone can be fixed by screws or Kirschner wires alone, which can then be protected by a transarticular external fixator for a short period of time, thus reducing the influence on activities of adjacent joints. Second, FVFG is appropriate when the infection site is large or the bone defects are long, especially for cases with an unclear range and margin of infected lesions on preoperative X-ray. When the infection site is too large, complete debridement is more difficult. In this situation, the IBT procedure is markedly prolonged, which can result in more severe complications, longer external fixation times, increased re-operation rate, and operation failure. Conversely, in FVFG, the fibula carrying vascular pedicles and attached muscle flaps can restore blood supply, reduce infection risk, and allow for the preservation of partial healthy cortical bone or soft tissues of the lesion. For relatively large bone defects, the residual bone segment with blood supply can be placed in parallel with the fibular graft, folded with the fibular flap, or transplanted with the iliac flap with or without blood vessels to increase the bone volume, which can greatly reduce the risk of stress fracture during healing [16]. El-Gammal et al. [12] reported that the Ilizarov technique has greater efficacy when the tibial defect length is less than 12 cm, whereas FVFG is more efficacious when the tibial defect length is 12 cm or more. Third, simultaneous grafting of fibular and skin flaps is recommended for patients with complicated soft tissue defects, and soft tissue defects can be repaired simultaneously to shorten the exposure time of deep tissues and reduce the recurrence rate of infection. Ozaksar et al. [24] reported mean bony union time for the proximal and distal fibula of 19 weeks (range, 16 to 24 weeks) following Gustilo type III open tibial fracture using this technique of carried flaps for repair of soft tissue defects. Bumbasirevic et al. [27] reported that viable pedicle anastomoses can be obtained using skin islands/pedicles as large as 10 cm × 20 cm. In the current study, the largest fibular flap used was 8 cm × 15 cm. For patients with larger wounds, a free flap was initially utilized for wound repair, followed by repair and reconstruction of the femoral/tibial defects in the second stage. Indeed, multiple techniques are often required in combination for specific cases. For example, Semaya et al. [28] treated 40 patients with tibial bone defects using combined FVFG and IBT to achieve high clinical efficacy as indicated by the average recovery time of 7.3 months (range, 6–12 months) until unprotected full weight-bearing.

The final limb function, especially the range of motion of knee/ankle joint, is closely related to bone healing time and external frame fixation time. In the FVFG group, it can provide blood supply for bone healing, reduce bone healing time, reduce external frame fixation time, which provide good conditions for the exercise of peripheral joints. While in the IBT group, it needs a longer time for bone transfer, and soft tissue is cut and pulled during bone transport. Especially in transportation of the femur, it has great influence on the thigh muscles, which can easily lead to muscle injury or contracture and lead to postoperative knee stiffness. From this point of view, if patients with femoral infectious bone defect were made treatment with FVFG, it will have certain advantages for postoperative knee function recovery.

In this case series, the fibular graft was smaller than the femur/tibia at the recipient site, so we modified the standard methods by increasing bone volume to prevent fracture of the fibular graft union. In addition, the external fixation time was appropriately prolonged after fibular grafting to avoid premature and excessive weight-bearing. Previous studies have reported that certain postoperative complications occur in the donor, although a majority of patients have no long-term functional limitations [27, 29, 30]. For instance, Garrett et al. [31] found that preservation of 8–10 cm of the distal fibular length resulted in no significant donor-site morbidity, and Pacelli et al. [32] found that preserving 10% of the residual distal fibular length was sufficient to maintain ankle stability. Similarly, fibular osteotomy was performed at 10 cm or above the distal fibular length in the current study, with extensive protection of the superficial peroneal nerve branch during the operation. Moreover, the ankle joint was fixed in the neutral position with braces for 3 weeks postoperatively. Aside from slightly reduced muscular strength for a short period, no other complications occurred, and muscle strength was restored at 6 months post-surgery.

For patients receiving IBT, the external fixator with transport function was selected according to the lower extremity defect site(s). A unilateral external fixator is often recommended in clinical practice since it is more comfortable to carry [33–35]. When the external fixator is adopted, the correct mechanical angle of the lower extremity must be maintained to avoid angulation and rotation. In addition, important blood vessels and nerves must be protected from injury by fixation nails. To prevent sliding at the proximal or distal fibula and ensuing effects on joint stability, nails (or needles) can be placed at the proximal or distal tibia and fibula for simultaneous fixation. In this study, IBT was initiated at 1 week after osteotomy. The distraction rate was 0.5–1 mm/day and distraction was performed 2–4 times/day. The sliding speed was adjusted according to the osteotomy site, age, and postoperative osteogenic capacity as assessed by X-ray. Limb shortening is frequently employed to reduce the wound size and promote earlier contact between bone ends. In general, acute limb shortening should not exceed 4–6 cm. Also, the peripheral blood supply of the extremities should be closely monitored to prevent limb sensory disturbance or ischemic necrosis caused by the overlap and tortuosity of nerves and blood vessels. Eventually, the shortened limbs can be slowly lengthened by the second-stage Ilizarov technique [3, 36]. Wu et al. [3] reported similar outcomes using IBT or bone shortening–lengthening for tibial bone and soft tissue defects, but the latter required less union time and promoted faster weight-bearing. Zhang et al. [37] applied double-level IBT for the treatment of large post-traumatic tibial defects and found it to be a safe and reliable method to reduce bone transport and in-frame time.

It should be emphasized that FVFG and IBT are only reconstruction techniques (together with the Masquelet technique) and are not intended to cure the infection. Infection treatment is with bone debridement. A variety of techniques are needed for debridement, in order that the infection would be controlled [38]. Clinically, the importance of “early debridement, multiple debridement and thorough debridement” is generally recognized. This is thought that it is easier to treat the limb with tissue defect than to treat the infected limb.

## Conclusions

Taken together, the repair and reconstruction of infected bone defects in the lower extremities (femur/tibia), especially cases with complicated soft tissue defects, are extremely challenging and require prolonged treatment and recovery. Both FVFG and IBT are effective treatments for infected bone defects, each with specific advantages and disadvantages depending on individual clinical conditions. We recommend FVFG for patients with complicated soft tissue defects, bone defects adjacent to joints, and large bone defects, especially unicortical defects, and for patients able-to-tolerate microsurgery. On the contrary, IBT should be considered for patients with poor soft tissue conditions surrounding the extremities, poor vascular quality, and multiple injuries.

## Data Availability

None

## Abbreviations

FVFG: Free vascularized fibular graft
IBT: Ilizarov bone transport
